# Multifunctional regulation of VAMP3 in exocytic and endocytic pathways of RBL-2H3 cells

**DOI:** 10.3389/fimmu.2022.885868

**Published:** 2022-08-05

**Authors:** Satomi Mishima, Marin Sakamoto, Hikaru Kioka, Yuka Nagata, Ryo Suzuki

**Affiliations:** Faculty of Pharmaceutical Sciences, Institute of Medical, Pharmaceutical and Health Sciences, Kanazawa University, Kanazawa, Japan

**Keywords:** allergy, mast cell (MC), SNAREs, VAMP3, FcϵRI, exocytosis

## Abstract

Mast cells (MCs) are inflammatory cells involved in allergic reactions. Crosslinking of the high-affinity receptor for IgE (FcϵRI) with multivalent antigens (Ags) induces secretory responses to release various inflammatory mediators. These responses are largely mediated by soluble N-ethylmaleimide-sensitive factor attachment protein receptors (SNAREs). Vesicle-associated membrane protein 3 (VAMP3) is a vesicular-SNARE that interacts with targeted SNARE counterparts, driving the fusion of MC secretory granules with the membrane and affecting subsequent assembly of the plasma membrane. However, the role of VAMP3 in FcϵRI-mediated MC function remains unclear. In this study, we comprehensively examined the role of VAMP3 and the molecular mechanisms underlying VAMP3-mediated MC function upon FcϵRI activation. VAMP3 shRNA transduction considerably decreased VAMP3 expression compared with non-target shRNA-transduced (NT) cells. VAMP3 knockdown (KD) cells were sensitized with an anti-DNP IgE antibody and subsequently stimulated with Ag. The VAMP3 KD cells showed decreased degranulation response upon Ag stimulation. Next, we observed intracellular granule formation using CD63-GFP fluorescence. The VAMP3 KD cells were considerably impaired in their capacity to increase the size of granules when compared to NT cells, suggesting that VAMP3 mediates granule fusion and therefore promotes granule exocytosis in MCs. Analysis of FcϵRI-mediated activation of signaling events (FcϵRI, Lyn, Syk, and intracellular Ca^2+^ response) revealed that signaling molecule activation was enhanced in VAMP3 KD cells. We also found that FcϵRI expression on the cell surface decreased considerably in VAMP3 KD cells, although the amount of total protein did not vary. VAMP3 KD cells also showed dysregulation of plasma membrane homeostasis, such as endocytosis and lipid raft formation. The difference in the plasma membrane environment in VAMP3 KD cells might affect FcϵRI membrane dynamics and the subsequent signalosome formation. Furthermore, IgE/Ag-mediated secretion of TNF-α and IL-6 is oppositely regulated in the absence of VAMP3, which appears to be attributed to both the activation of FcϵRI and defects in VAMP3-mediated membrane fusion. Taken together, these results suggest that enhanced FcϵRI-mediated signal transduction in VAMP3 KD cells occurs due to the disruption of plasma membrane homeostasis. Hence, a multifunctional regulation of VAMP3 is involved in complex secretory responses in MCs.

## Introduction

Mast cells (MCs) possess specialized secretory granules, which fuse with the plasma membrane upon immunoglobulin E-receptor (IgE-FcϵRI) stimulation with multivalent antigens (Ags), thereby releasing inflammatory mediators (1). During the exocytotic process (e.g., degranulation), secretory granules fuse with each other prior to contact with the plasma membrane. A family of membrane fusion proteins called soluble N-ethyl-maleimide-sensitive factor attachment protein receptors (SNAREs), which are present on both the granule and the plasma membrane, can drive granule secretion by mediating either granule-to-plasma membrane fusion or granule-to-granule fusion (2). This also affects subsequent membrane assembly and remodeling processes. Thus, MC degranulation and retrieval are largely mediated by SNARE proteins (3, 4).

Vesicle-associated membrane protein 3 (VAMP3) is a ubiquitously expressed vesicular-SNARE that interacts with its targeted SNARE counterparts, such as syntaxin homologs 4 or 13 and soluble N-ethylmaleimide-sensitive fusion protein 23 (SNAP23), leading to vesicle fusion and exocytosis (5, 6). In human synovial sarcoma cells, which are synoviocyte models for extensive studies of rheumatoid arthritis and produce inflammatory cytokines (7), VAMP3 and SNAP23 contribute to the IL-1β-induced Ca^2+^-dependent secretion of inflammatory cytokines (8). Boddul et al. have demonstrated that VAMP3 and SNAP23, but not syntaxin homologs 2, 3, or 4, are involved in the exocytosis of cytokines in inflamed synoviocytes, highlighting the functional importance of VAMP3 and SNAP23 in the exocytosis pathway of inflammatory cytokines (8). In addition, VAMP3, which is present in professional phagocytes such as macrophages, is also associated with the membrane trafficking pathway linking cytokine secretion and phagocytosis of pathogens (9). This association may economize membrane transport and augment the immune response through the delivery of cytokine from the Golgi to the recycling endosome, where VAMP3 is described as a reliable marker of recycling endosomes (9, 10), which would be further discussed in the discussion section. Therefore, VAMP3 and its partner SNAREs are involved in both exocytosis and endosomal recycling. However, these effects are likely to be observed mainly in inflamed tissue models and phagocytes in macrophages (11, 12). Whether it also operates in MCs, particularly the mechanism by which VAMP3 functions in Ag-bound IgE and FcϵRI-mediated activation, which is a typical allergic response triggered by MCs, has not been fully elucidated.

Secretory granules in a wide range of hematopoietic cells, including MCs, contain a variety of biologically active inflammatory mediators, such as histamine, proteases, cytokines, and leukotrienes. These mediators are recognized to be differentially packed in different types of secretory granules, and SNAREs are strongly associated with the secretion of distinct granules (1, 13). For example, in the context of the mobilization of human neutrophil granules, which is critical for the innate immune response, Mollinedo et al. reported that specific and tertiary granules that are readily exocytosed upon cell activation contained VAMP1, VAMP2, and SNAP23, whereas azurophilic granules that are mainly mobilized to the phagosome were enriched in VAMP1 and VAMP7. Syntaxin4 in the plasma membrane was indicated to mediate the secretion of both specific/tertiary and azurophilic granules. They performed ultrastructural, co-immunoprecipitation, and functional assays and suggested that at least two SNARE complexes, made up of syntaxin4/SNAP23/VAMP1 and syntaxin4/SNAP23/VAMP2, are involved in the exocytosis of specific and tertiary granules, whereas interactions between syntaxin4 and VAMP1/VAMP7 are involved in the exocytosis of azurophilic granules (14). Furthermore, Puri et al. found that deletion of VAMP8 in MCs profoundly inhibited FcϵRI-mediated exocytosis of serotonin and cathepsin D, but had no effect on either histamine or TNF-α release (1). Therefore, although it has been suggested that functionally distinct secretory granule subsets whose function is regulated by different SNARE proteins exist in MCs, considerable uncertainty remains regarding the precise characterization and fusion of each distinct granule subtype and SNARE involvement (1, 15).

Elucidating the characteristic features of SNAREs, including VAMP3, may contribute to understanding the molecular mechanisms underlying MC activation and mediator secretion in response to Ag. Herein, we comprehensively studied the role of VAMP3 in MCs using the VAMP3 knockdown approach in RBL-2H3 cells, a model for MC IgE-mediated responses.

## Materials and methods

### Cell culture

RBL-2H3 cells were cultured in RPMI 1640 (Nacalai Tesque, Tokyo, Japan) supplemented with 10% FBS, 2 mM L-glutamine, penicillin (100 IU/ml), and streptomycin (100 IU/ml) at 37°C in a humidified 5% CO_2_ atmosphere. Cells were sensitized with anti-DNP IgE (1 μg/ml) and stimulated with DNP-HSA (50 ng/ml; Sigma-Aldrich, St Louis, MO, USA). The β-hexosaminidase release assay was performed according to an established method (16). Briefly, the collected supernatant was incubated with *p*-nitrophenyl-N-acetyl-β-D-glucosaminide (2 mM) dissolved in 0.1 M citrate buffer (pH 4.5) for 1 h at 37°C. 0.1 M carbonate buffer (pH 10) was then added to the reaction wells to stop the reaction and develop color. Colorimetric measurements were performed at a wavelength of 405 nm using the Tecan Infinite F50 microplate reader (Tecan, Mannedorf, Switzerland).

### Lentivirus infection

Clones of RBL-2H3 cells with stable knockdown of VAMP3 expression were generated using a commercial lentiviral system for the introduction of short hairpin RNAs (shRNAs; Sigma, St. Louis, MO, USA). Sigma Mission non-target shRNA (SHC002) was used to generate the control cell line, and shRNA TRCN0000110516 was used to knockdown VAMP3 expression. Lentiviral particles were added to RBL-2H3 cells for 1 week, and the cells were subsequently cultured in medium containing 1.5 μg/ml puromycin. CD63-GFP and RFP-VAMP3 were transiently transfected by electroporation (CUY21EditII, BEX, Tokyo, Japan) using the pTagGFP2-N and pTagRFP-C vectors. Fluorescent images were obtained using a confocal laser-scanning microscope (LSM710, Carl Zeiss, Oberkochen, Germany). The primer sets used for the construction of GFP-tagged CD63 and RFP-tagged VAMP3 are listed in **Supplementary Table 1**. Details of the plasmid construction are described in the **Supplementary Experimental Procedures**.

### Immunoblotting

Cells were harvested and lysed in lysis buffer containing protease inhibitors and centrifuged at 14,000 × g for 10 min. The supernatants were mixed with SDS loading buffer followed by heating at 95°C for 5 min and subjected to SDS-PAGE. Membranes were blocked with blocking buffer (BlockPRO Protein-Free Blocking Buffer, Visual Protein Biotechnology Corporation, Taipei, Taiwan) and Western blotting was performed using anti-phospho-FcϵRIγ (Y47, clone: γ-pY^47^) which was produced as previously described (17) and provided as a kind gift from Dr. Juan Rivera from the US National Institutes of Health, anti-phospho Lyn (Y396), anti-phospho Syk (Y519 and Y520), anti-phospho LAT (Y191), anti-phospho Akt (T308), anti-phospho Erk1/2 (T202 and Y204), anti-phospho p38 (T180 and Y182), anti-VAMP3 (clone: N-12, Santa Cruz Biotechnology, Santa Cruz, CA, USA), anti-FcϵRβ (clone: JRK), which was a kind gift from Dr. Juan Rivera from the US National Institutes of Health (18), and anti-β-actin (MBL, Nagoya, Japan), followed by staining with the appropriate secondary antibodies. For the immunoprecipitation of FcϵRI, supernatants were incubated with goat anti-mouse IgE (Southern Biotech, Birmingham, AL, USA) for 1 h at 4°C followed by incubation with protein A-Sepharose beads (GE Healthcare, Chicago, IL, USA) for 1 h at 4°C. Beads were washed two times with lysis buffer and boiled in SDS sample buffer at a non-reducing condition for 5 min. Images were scanned using Odyssey (LiCOR Biosciences, Lincoln, NE, USA). Band intensity was measured using ImageJ software (NIH Image, Bethesda, MD, USA). Each band intensity was normalized to that of the internal control, and the data are presented as a relative fold change. None of the images shown were modified using nonlinear adjustments.

### Immunostaining

Anti-VAMP3 (Proteintech, Chicago, IL) followed by appropriate secondary antibodies, FITC-labeled anti-TNFα (BioLegend, San Diego, CA), and APC-labeled anti-IL-6 (BioLegend, San Diego, CA) were used for immunocytochemistry. The cells were fixed with 4% paraformaldehyde and permeabilized with 0.2% Triton X-100 prior to staining. Fluorescent images were obtained by confocal laser scanning microscopy (CLSM; LSM 710 Carl Zeiss, Oberkochen, Germany). Anti-FcϵRIα (Merck Millipore, Temecula, CA, USA) followed by the appropriate secondary antibody and FITC-labeled anti-IgE (BD Biosciences, San Jose, CA) were used for fixed and unpermeabilized cells. Flow cytometry was performed on a FACSVerse flow cytometer (BD Biosciences, San Jose, CA). The fluorescence intensity was analyzed using ImageJ software (NIH Image) and MIPAV software (NIH Image). None of the images shown were modified using nonlinear adjustments.

### Flow cytometry

The stimulated cells were fixed with 4% paraformaldehyde. Anti-IgE-fluorescein isothiocyanate (FITC, BD Biosciences) was used for labeling the fixed/non-permeabilized cells. Flow cytometry was then performed on a FACSVerse flow cytometer (BD Biosciences).

### Intracellular Ca^2+^ measurements

IgE-loaded cells were incubated with 2 μM Fluo-4 acetoxymethyl ester (AM, Dojindo Laboratories, Kumamoto, Japan) and 10 μM Fura Red AM (Invitrogen, Carlsbad, CA, USA) for 30 min. The cells were then washed and resuspended in Tyrode’s buffer (1.8 mM CaCl_2_). An LSM710 CLSM was used for ratiometric measurements with 488 nm wavelength light from an Ar laser. The fluorescence of Fluo-4 and Fura Red was detected using a bandpass filter (505–530 nm) and a longpass filter (>560 nm), respectively. Calcium responses were calculated as the ratio of Fluo-4 to Fura Red, normalized as a fold increase relative to the signal in unstimulated cells. Fluorescence images were collected every 5 s, and fluorescence intensity was quantified using LSM 710 software.

### Quantitative RT-PCR

The cells were lysed using TRIzol reagent (Invitrogen, Carlsbad, CA, USA). cDNA was synthesized using an RT Reagent Kit (Toyobo, Osaka, Japan). Real-time RT-PCR was performed using SYBR Green Master Mix (Luna Universal qPCR master mix, New England BioLabs, Ipswich, MA, USA) and analyzed using the MX3005P qPCR System (Stratagene, La Jolla, CA, USA). Gene expression was normalized to the internal control *GAPDH* gene, and the results were expressed as fold stimulation over control. Primer sequences used are listed in **Supplemental Table 2**.

### Enzyme-linked immunosorbent assay (ELISA)

The cell supernatants were collected after 3 h (TNF-α) and 9 h (IL-6) of stimulation with DNP-HSA. The release of TNF-α and IL-6 was determined using an ELISA kit (R&D Systems, Minneapolis, MN, USA), according to the manufacturer’s instructions. Briefly, supernatants of the samples were placed into 96-well plates coated with anti-rat TNF-α or anti-rat IL-6 antibodies after blocking the Assay Diluent buffer. The plates were washed five times and biotinylated detection antibodies and streptavidin-HRP were added. This was followed by recording the absorbance (optical density (OD)) using a microplate reader.

### Statistical analysis

Statistical significance was assessed using GraphPad Prism, version 7.0d (GraphPad Software, San Diego, CA, USA). Statistical differences were determined using Student’s t-test (between two groups), one-way analysis of variance (ANOVA) followed by Tukey’s or Dunnett’s multiple-comparison test as appropriate (for comparing multiple groups or time-dependent changes), or two-way ANOVA (for curves). Statistical significance was set at p < 0.05.

## Results

### VAMP3 is involved in the granule-to-granule membrane fusion in IgE/Ag-activated MC

**Figure 1:**
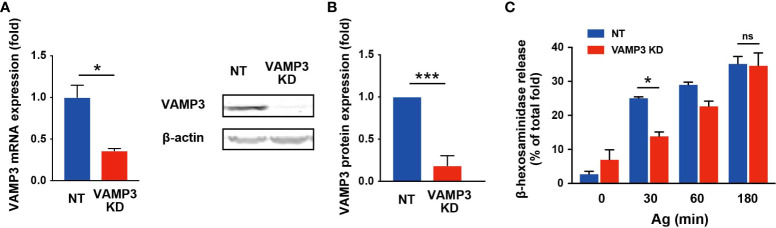
VAMP 3 knockdown attenuates β-hexosaminidase release in response to Ag VAMP3-stable KD RBL-2H3 cells were stimulated with IgE/Ag (50 ng/ml). **(A)** VAMP3 expression was analyzed using RT-PCR. **(B)** Expression of VAMP3 protein was confirmed using western blotting. The bar graph shows band density normalized to the loading control and compared to that of NT cells. **(C)** Released β-hexosaminidase was measured in the supernatant and represented as a percentage of total β-hexosaminidase in the cells. NT: control knockdown with a non-targeting shRNA plasmid. Ag: antigen. Data are presented as the mean ± standard error. n = 3 independent experiments **(A–C)**. *p<0.05, ***p<0.001; ns, not significant.

**Figure 2:**
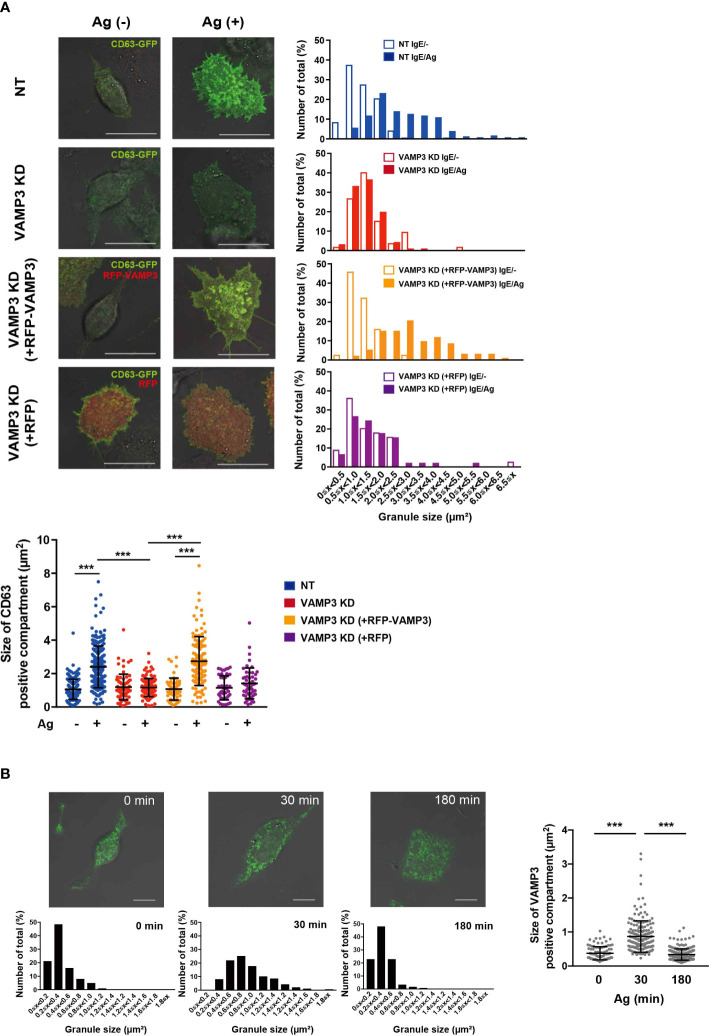
VAMP3 mediates granule-to-granule fusions during granule exocytosis VAMP3-mediated granule-to-granule fusion was analyzed using confocal laser scanning microscopy. **(A)** Cultured cells were transfected with GFP-tagged CD63 and/or RFP-tagged VAMP3 and stimulated with Ag (50 ng/ml) for 30 min. Bar graphs show size distribution of the CD63 positive granule; representative images are shown from three independent experiments (magnification ×63, scale bar = 20 μm). Dot plot represents the average size of granules in all analyzed events (granules) counted from 47–236 granules from 3–5 cells/sample from 3 independent experiments. **(B)** IgE/Ag stimulated RBL-2H3 cells were stained for VAMP3. Green indicates Alexa 488-stained VAMP3. Bar graphs represent size distribution of the VAMP3 positive granule analyzed; representative images are shown from three independent experiments (magnification ×63, scale bar = 10 μm). Dot plot represents the average size of granules in all analyzed events (granules) counted from 101–235 granules from 3–5 cells/sample from three independent experiments. NT: control knockdown with a non-targeting shRNA plasmid. Ag: antigen. ***p<0.001.

To explore the role of VAMP3 in MCs, MC functions were analyzed in VAMP3 knockdown (KD) cells in response to Ag stimulation. VAMP3 knockdown (KD) in MCs was performed using shRNA transfection. The VAMP3 KD cells showed a significant decrease in VAMP3 expression at both the mRNA and protein levels (**Figures 1A, B**). Additionally, we analyzed the off-target effects in VAMP3 KD cells; the expression levels of VAMP7, which is a SNARE family member and has an important function in MC secretory response (19), were not altered in VAMP3 KD cells when compared to that in NT cells (**Supplementary Figure 1**). The MC degranulation response following Ag stimulation was analyzed by measuring the granule enzyme β-hexosaminidase, which is commonly used as a biomarker of MC degranulation. A time-dependent increase for the β-hexosaminidase secretion, which peaked at 30–60 min following Ag stimulation has previously been reported; therefore, the secretion of preformed mediator is generally assessed within 30–60 min of Ag stimulation (20). Herein, the degranulation response was significantly attenuated in VAMP3 KD cells compared to that in non-targeted shRNA (NT) transduced cells at 30 min after Ag stimulation (**Figure 1C**). However, when we observed the degranulation response 180 min after Ag stimulation, there were no significant differences between NT and VAMP3 KD cells (**Figure 1C**). There was a gradual increase in the Ag-induced β-hexosaminidase release in VAMP3 KD cells compared to that in NT cells, which represented a rapid sigmoidal increase. These findings indicate that while VAMP3 indeed contributes to the Ag induced rapid secretion of β-hexosaminidase, certain compensatory or redundant mechanisms appear to be operating at the later time point of the secretion in MC, consistent with previous reports (4, 21). The β-hexosaminidase release in the VAMP3-rescued VAMP3 KD cells was not restored, since the transient expression of VAMP3 in VAMP3 KD cells is limited to a certain population by its low transfection efficiency. Although whole cell population analysis for the β-hexosaminidase release assay was insufficient to estimate the restoration of VAMP3 in our experimental setting, microscopic observation or FACS analysis at single cell levels enabled the analysis of the VAMP3 KD phenotype rescue. To further explore the role of VAMP3 in MC degranulation, we explored granule fusion in VAMP3 KD cells by monitoring the secretory granule marker CD63, which demonstrated that VAMP3 deficiency did not increase granule size, even after Ag stimulation, and this was rescued by the ectopic expression of RFP-VAMP3 positive granules (**Figure 2A**). In the gain of function experiments, FcϵRI surface expression was restored in VAMP3-rescued VAMP3 KD cells (**Supplementary Figure 2**). Furthermore, we assessed the size of secretory granules expressing VAMP3 in wild-type RBL-2H3 cells by immunostaining with an anti-VAMP3 antibody. As shown in **Figure 2B**, the size of the VAMP3 positive compartment showed a significant increase 30 min after Ag stimulation, indicating that VAMP3-mediated granule-to-granule fusion occurred during MC degranulation. This increase in size was no longer observed 180 min after Ag stimulation, and the granule size returned to the prestimulation level (**Figure 2B**). These observations indicate that VAMP3 mediates Ag-induced granule-to-granule fusion during degranulation, thereby contributing to robust degranulation responses in MCs.

### VAMP3-deficiency enhances IgE/Ag-induced MC signaling

**Figure 3:**
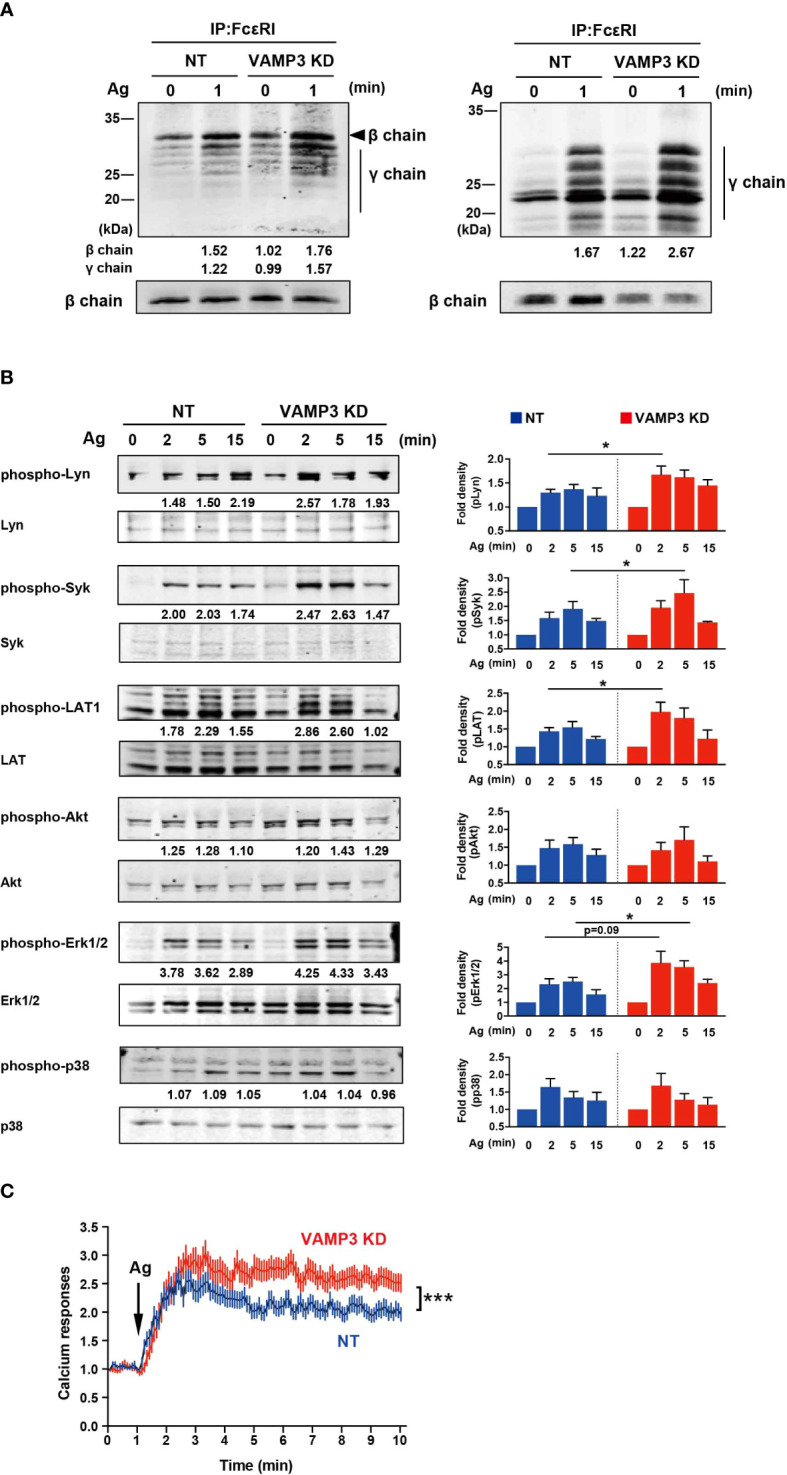
FcϵRI phosphorylation is upregulated following Ag stimulation FcϵRI signaling following Ag stimulation (50 ng/ml) was analyzed, which resulted in enhanced VAMP3 KD. **(A)** FcϵRI was precipitated and analyzed by immunoblotting with antibodies specific for tyrosine-phosphorylated proteins (4G10, left panel) and phosphorylated ITAMs of FcϵRIγ (right panel). Expression of β-chain was measured as an internal loading control by re-blotting the membranes with anti-β chain antibody JRK. **(B)** A series of protein phosphorylation events triggered by FcϵRI crosslinking with the Ag was analyzed. **(C)** Calcium response. Each line represents the mean ± standard error of 51-69 single cells/group from 4 independent experiments. One representative blot of three independent experiments is shown. The band density normalized to that of unstimulated cells is shown below each band **(A, B)**. The P values refer to the differences between two groups that were analyzed using the two-tailed Student’s t-test. NT: control knockdown with a non-targeting shRNA plasmid. Ag: antigen. *p<0.05, ***p<0.001.

SNAREs are responsible not only for the exocytotic granule-to-plasma membrane and granule-to-granule fusion, but also contribute to endocytic membrane recycling (12, 22). Thus, VAMP3 KD cells have been hypothesized to be related to the deficiency of plasma membrane assembly. The Lucifer Yellow uptake experiment, which measures endocytosis of Lucifer Yellow dye, clearly demonstrated a dysfunction in endocytosis in VAMP3 KD cells (**Supplementary Figure 3**). This loss of plasma membrane homeostasis is thought to be reflected by altered FcϵRI receptor function in response to exogenous Ag. FcϵRI receptor consists of heterotetramer αβγ2-chains, and in response to Ag, Lyn kinase phosphorylates the ITAMs of FcϵRIβ (βITAM) and FcϵRIγ (γITAM), which initiates a complex signaling cascade involving a series of membrane-associated and cytoplasmic signaling molecules (23, 24). As shown in **Figure 3A**, FcϵRI in MCs was precipitated with an anti-IgE antibody and subjected to immunoblot analysis. Tyrosine phosphorylation of ITAM in the FcϵRIβ- and γ-signaling subunits was detected with the anti-phosphotyrosine antibody (clone 4G10), as represented in the left panel in **Figure 3A**; the phosphorylated Y47 site of ITAM within FcϵRIγ is represented in the right panel. The antibody γ-pY^47^ allows the detection of the phosphorylated γ-chain ITAM Y47. The amount of FcϵRI β chain used as loading control was detected using anti-FcϵRIβ antibody (clone JRK). Tyrosine phosphorylation in both βITAM and γITAM was significantly enhanced in VAMP3 KD cells (**Figure 3A**). Consistently, subsequent FcϵRI signaling molecules including Lyn, Syk, LAT, and ERK1/2 were also significantly activated in the VAMP3 KD cells compared to that in NT cells (**Figure 3B**). The average Akt and p38 phosphorylation band densities were higher in VAMP3 KD cells compared to that observed in NT cells at the peak time point of phosphorylation. Furthermore, intracellular signaling with Ca^2+^ as a second messenger is important for MC activation, and we observed an upregulation in cellular Ca^2+^ signal propagation following Ag stimulation. VAMP3 KD cells showed a significant and sustained upregulation of Ca^2+^ signaling throughout the observation period (**Figure 3C**).

### VAMP3 regulates surface expression of FcϵRI and membrane homeostasis

**Figure 4:**
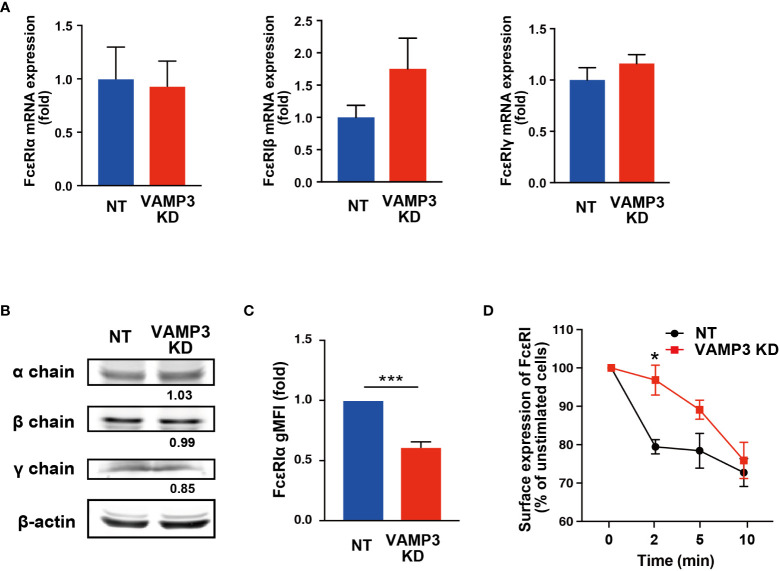
No FcϵRI upregulation was observed in cell surface expression or internalization The expression of each subunit of FcϵRI in VAMP3 KD cells was determined. **(A)** Gene expression was analyzed using quantitative RT-PCR. **(B)** Protein expression was analyzed using western blot analysis. **(C)** Cell surface expression levels of FcϵRIα that binds to the IgE Fc region were analyzed using flow cytometry in the cells at steady state. **(D)** Ag-induced time-dependent reduction in cell surface FcϵRI-bound IgE was analyzed using flow cytometry. A representative blot is shown for three independent experiments, and the band density normalized to that of unstimulated cells is shown below each band **(B)**. Data are presented from three independent experiments. Three biological repeats were quantified in a single run of PCR in the qPCR analysis. NT: control knockdown with a non-targeting shRNA plasmid. Ag: antigen. gMFI: geometric mean fluorescence intensity. *p<0.05, ***p<0.001; ns, not significant.

The mechanism by which plasma membrane dysfunction caused by VAMP3 elimination led to enhanced FcϵRI activation remained unclear. To address this issue, we examined whether a quantitative and/or locational difference existed in FcϵRI between NT and VAMP3 KD cells. The total cellular expression of FcϵRI α, β, and γ chains did not differ between NT cells and VAMP3 KD cells at both the mRNA and protein levels (**Figures 4A, B**). Unexpectedly, the cell surface expression of FcϵRI showed a significant decrease in VAMP3 KD cells (**Figure 4C**), despite the cells displaying augmented FcϵRI signaling (**Figure 3** **)**. In addition, FcϵRI clustering and subsequent internalization in response to IgE/Ag were observed (**Figure 4D**). The surface IgE-FcϵRI complex was monitored using FACS, which showed a slow rate of internalization of FcϵRI in VAMP3 KD cells for up to 5 min (**Figure 4D**). The slow rate of FcϵRI internalization in VAMP3 KD cells existed despite demonstrating augmented Ag-induced FcϵRI signaling (**Figure 3**). Given these contradicting results, we explored the total number of cellular lipid rafts in MCs. Using confocal laser scanning microscopy, a lipid raft marker ganglioside GM1 was detected with the cholera toxin subunit B. Results revealed an enhanced amount of the ganglioside GM1 in VAMP3 KD cells indicating a significant increase in the number of lipid rafts upon VAMP3 elimination (**Supplementary Figure 4**). Lipid raft enrichment in VAMP3 KD cells might be associated with the enhancement of FcϵRI phosphorylation following Ag stimulation as increase in GM1 ganglioside in the plasma membrane is responsible for regulating the activities of several membrane receptors (25, 26). Furthermore, lipid raft microdomains might contribute to FcϵRI clustering and its association with signaling molecules in the plasma membrane (27, 28). Based on these observations, we suggest that the enhanced activation of FcϵRI in VAMP3 KD cells might occur due to FcϵRI cluster formation in the lipid raft-modified membrane. Although further examination is required, these findings suggest a bifunctional role of VAMP3 in plasma membrane homeostasis and the subsequent activation of MCs.

### VAMP3 differentially regulates secretory responses of heterogeneity granules

**Figure 5:**
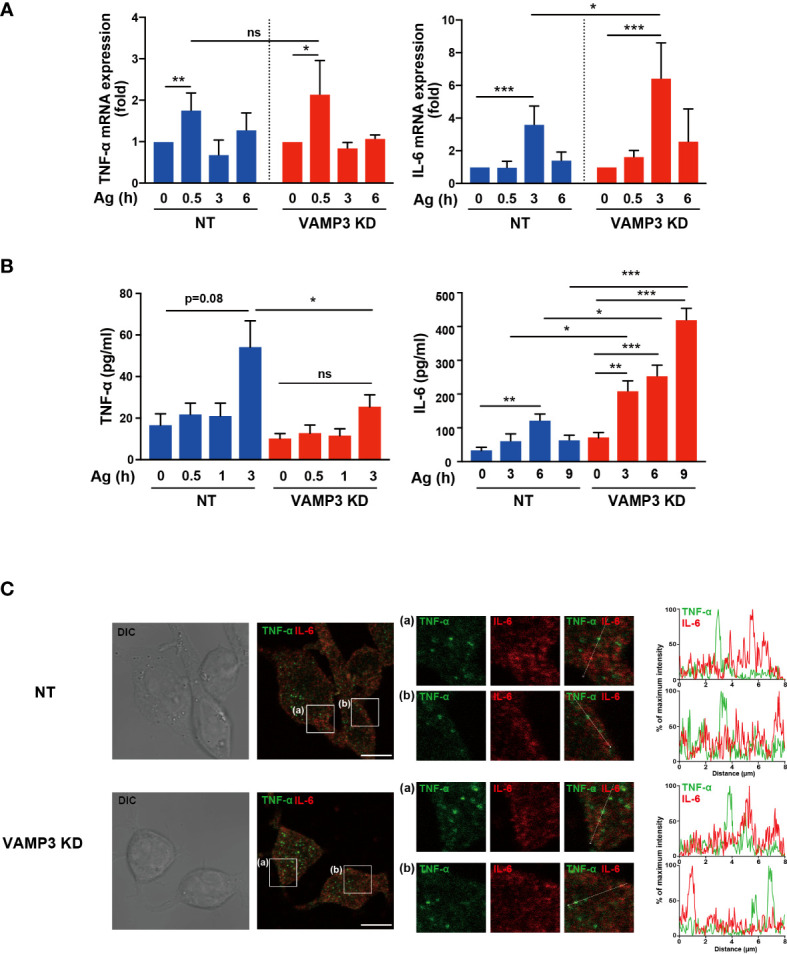
Ag-induced release of cytokines are differentially regulated by VAMP3 Ag stimulation (50 ng/ml)-induced cytokine expression and extracellular secretion were analyzed in the cells. **(A)** Time-dependent expression of TNF-α and IL-6 following Ag stimulation were compared to NT cells and VAMP3 KD cells. **(B)** Time-dependent secretion of TNF-α and IL-6 following Ag stimulation were analyzed. The supernatant of each cell type was subjected to ELISA. The qPCR data are presented from three to five independent experiments; three to five independent runs of PCR were performed. **(C)** Intracellular localization of TNF-α and IL-6 were analyzed using confocal microscopy. The large panels show transmitted light image (DIC) and merge image of TNF-α (green) and IL-6 (red) signals. The small panels **(a, b)** are magnified images of the area within the white square in the large panels. Representative images (magnification ×63, scale bar = 10 μm) and line profiles of fluorescence intensity were obtained from three independent experiments. Time-dependent differences were statistically validated using one-way ANOVA followed by multi-group comparisons. Differences between two groups were analyzed using the two-tailed Student’s t-test. NT: control knockdown with a non-targeting shRNA plasmid. Ag: antigen. *p<0.05, **p<0.01, ***p<0.001; ns, not significant.

As discussed above, VAMP3 plays multifunctional roles in membrane homeostasis and signaling events in MCs. To further examine the consequences of VAMP3-mediated regulation of MC signaling, we assessed IgE/Ag-induced cytokine expression and secretion in MCs. IgE/Ag-induced degranulation, which is monitored by β-hexosaminidase release, was significantly attenuated at 30 min in VAMP3 KD cells. This might have occurred due to the granule-to-granule fusion in the degranulation responses. However, a series of FcϵRI signaling events following IgE/Ag stimulation was augmented in VAMP3 KD cells. To address the complex regulation of MC functions, we analyzed the mRNA expression of several inflammatory mediators and secretory responses in VAMP3 KD cells. The tested inflammatory cytokines appeared to be differentially regulated in the context of VAMP3 involvement. RT-PCR analysis of the Ag-stimulated cells revealed that the transcription levels of IL-6 were significantly upregulated in VAMP3 KD cells 3 h after Ag stimulation, whereas TNF-α did not show a marked difference (**Figure 5A**). In addition, IL-2 and IL-13, but not CCL2 and IL-4, were significantly upregulated in VAMP3 KD cells at the peak time point of the expression (**Supplementary Figure 5**). We further explored time-dependent changes of protein secretion levels of TNF-α and IL-6 following Ag stimulation. The cytokine secretion has been generally observed not only in case of preformed mediator secretion (granule stored), but also in case of the *de novo* synthesized mediators. As shown in **Figure 5B**, there was a considerably high and sustained secretion of IL-6 up to 9 h following Ag stimulation in VAMP3 KD cells. In contrast, no significant increases were observed in TNF-α in VAMP3 KD cells. VAMP3 elimination attenuated Ag-induced TNF-α secretion from MCs, which is consistent with the results of the β-hexosaminidase release assay (**Figure 1C**), whereas IL-6 secretion was significantly enhanced in the VAMP3 KD cells (**Figure 5B**). To investigate the cause of differences in secretory response, we evaluated the distribution of TNF-α and IL-6 in MC granules and found that they exhibited differential distribution (**Figure 5C**). These data suggest that each granule contains a specific cytokine. The upregulated transcription and extracellular secretion of IL-6 in VAMP3 KD cells appear to be attributed to the enhancement of FcϵRI signal transduction as shown in **Figure 3**, whereas the deficiency of membrane fusion in certain granules and the plasma membrane contributed to the attenuation of robust secretory responses (i.e., β-hexosaminidase, TNF-α). Collectively, these data suggest that a multifunctional regulation of VAMP3 is involved in the complex secretory responses of MCs.

## Discussion

We demonstrated that VAMP3 is involved in both IgE/Ag-triggered granule exocytotic process and plasma membrane endocytic mechanism in MCs. VAMP3-mediated granule-to-granule fusion appears to be limited to a distinct granule population, which indicates that VAMP3 could contribute to the heterogeneity of secretory responses in MCs.

VAMP3 KD cells showed a markedly decreased in degranulation responses at 30 min, but not at 180 min, as assessed by β-hexosaminidase release. β-hexosaminidase is a glycolytic enzyme that is commonly recognized as a major component of MC granules, and its secretion is widely used as a marker for MC degranulation triggered by IgE/Ag. For β-hexosaminidase secretion, a time- dependent increase has been reported, which peaked at 30–60 min following Ag stimulation (20); therefore, the secretion of preformed mediator is generally assessed within this time frame. Indeed, we found similar results, wherein VAMP3-mediated regulation of secretory responses was observed 30 min post Ag stimulation (**Figure 1**). Previous studies have demonstrated that a membrane receptor (an opioid receptor) was recycled within 90 min in an agonist-induced endocytosis in HEK293 cells (29). In MCs, the endocytic recycling under degranulation might be occurring in newly formed secretory granules within 180 min. In addition, Ag-induced enlargement of VAMP3 positive granules were restored to normal levels at 180 min post stimulation, which might be caused by the recycled and/or newly formed secretory granules. Therefore, VAMP3 appears to contribute to the degranulation responses in MCs by mediating granule-to-plasma membrane fusion as well as intracellular granule-to-granule fusion, as indicated in **Figure 2**. The rescue of VAMP3 KD phenotype expectedly restored the Ag-induced granule fusions; however, whether the Ag-induced β-hexosaminidase release was rescued by VAMP3 remains to be determined. This transient rescue established using shRNA may possibly be achievable in whole cell population.

The endogenous and exogenous VAMP3 could be downregulated by stably expressing the siRNA. Indeed, FACS analysis in **Supplementary Figure 2** demonstrated lower GFP-VAMP3 expression efficacy in VAMP3 KD cells [VAMP3 KD (+VAMP3 GFP)] compared with GFP-transfected VAMP3 KD cells [VAMP3 KD (+GFP)]. In this study, we could exclude the possibility for off-target effects of VAMP7 and MC signaling molecules. Development of RNAi-resistant version of VAMP3 would further validate this. For the roles of VAMP3 on the MC signal transduction, the VAMP3 KD cells displayed enhanced susceptibility to exogenous Ag exposure by which the FcϵRI signal events including activation of Src family kinases Lyn, and other critical molecules and messengers (i.e., Syk, LAT1, Akt, MAPKs, Ca^2+^) were significantly upregulated. Crosslinking of FcϵRI with multivalent Ag initiates a series of cellular biochemical events leading to activation, which comprises the rapid release of granule-stored mediators and delays the secretion of *de novo*-synthesized mediators. These processes are coordinated by a complex cascade of signaling events involving the activation and recruitment of signaling molecules and scaffolding proteins in each step (30). Thus, an increase in FcϵRI signaling is a relevant feature of the MC allergic response to Ag stimulation. In VAMP3 KD cells, however, the enhanced FcϵRI signals did not reflect the major degranulation response monitored by β-hexosaminidase release. This complicated observation, illustrated in **Figures 1**–**3**, prompted us to identify the multifunctional roles of VAMP3 in MC activation and secretion. Indeed, VAMP3 has several distinct roles in secretory proteins, exocytic/endocytic pathways, trafficking, and processing of membranes, as described above (8–10). Specifically, the functions of VAMP3 have been well investigated in macrophage phagocytes. VAMP3 mediates TNF-α secretion in macrophages in which VAMP3 delivers cytokine-positive granules to the cell surface at the site of phagocytic cup formation (9). Granular fusion in forming of the phagocytic cup simultaneously allows the rapid release of TNF-α and expands the membrane for phagocytosis (9). Likewise, VAMP3 function has been known to be required for the efficient phagocytosis of yeast (31, 32). These important SNARE complexes in macrophages also have been found to facilitate cell adhesion, spreading, and persistent cell migration on fibronectin through the delivery of VAMP3-positive membrane with its cargo, to expand the plasma membrane and participate in organizing a specialized adhesive structure called podosome, which plays an important role in macrophage migration (33, 34). Therefore, the role of VAMP3 in MCs is thought to be associated with both membrane endosome process and exocytotic granule release.

In this study, the cellular uptake of Lucifer Yellow dye, which is used to monitor endocytosis, demonstrated significant reduction in the level of endocytosis in VAMP3 KD cells compared to that in NT cells. This observation appears to be consistent with previous studies reporting the involvement of VAMP7 in mediating vesicular transport within the endosomal pathway. VAMP7 plays a role in early endosome to lysosome vesicular trafficking (35). A study using immunoelectron microscopy analysis showed that VAMP7 is primarily concentrated in the trans-Golgi network region of the cells, as well as in late endosomes and transport vesicles that do not contain the mannose-6 phosphate receptor, which is responsible in mediating the transport of newly synthesized lysosomal enzymes to endosomes (35). More recently, Tang BL reported that in autophagy, sytaxin16 and VAMP7 could be involved in the regulation of autophagosome formation and subsequent lysosomal fusion (36). Moreover, syntaxin17 phosphorylation regulates the initiation of autophagy (36, 37). Given these observations and our findings in the present study, further investigations of cell surface FcϵRI receptor expression and its functions in the context of internalization were conducted, revealing a significant reduction in MC surface FcϵRI in VAMP3 KD cells. Remarkably, in NT cells, FcϵRI internalization occurred immediately after Ag stimulation; however, in VAMP3 KD cells, FcϵRI internalization showed a significantly slower rate. Ag binding to IgE-bound FcϵRI causes cross-linking of IgE–FcϵRI complexes. FcϵRI dynamics on the plasma membrane determine FcϵRI-mediated signal transduction (38). FcϵRI behavior in the plasma membrane of MCs has been suggested to correlate with FcϵRI signal initiation (39, 40). According to these observations, the slower rate of IgE-FcϵRI internalization observed in VAMP3 KD cells suggest that the IgE-FcϵRI complex remained on the cell surface in VAMP3 KD cells longer compared to that in NT cells and/or altered its aggregation behavior to a certain degree in VAMP3 KD cells, and this was associated with the enhanced phosphorylation of FcϵRI in response to Ag.

To further examine the mechanism underlying the induction of FcϵRI activation and subsequent signaling upon VAMP3 elimination, we analyzed lipid raft formation in MCs and observed a considerable increase in the lipid raft domain in VAMP3 KD cells. Lipid rafts are ordered regions of the plasma membrane that are enriched in cholesterol and glycosphingolipids, which play important roles in receptor-mediated signaling (41, 42). Thus, the integrity of lipid rafts significantly impacts cell surface receptor stabilization and is associated with signaling molecules in the membrane. Indeed, in MCs, lipid rafts are crucial for FcϵRI function and actin cytoskeleton reorganization (43, 44). Furthermore, lipid raft stability is known to be capable of enhancing FcϵRI signaling, possibly by suppressing phosphatase activity, where the lipid raft submembrane location is critical to phosphatase-mediated regulation of Lyn kinase activity that supports activation of FcϵRI (28). A more recent study showed that lipid rafts form transient proteins that are protected while in a raft and are subject to dephosphorylation when the raft breaks up; such proteins are found in the non-raft region of the membrane (27, 45). Based on these observations, our findings suggest that the enhancement of lipid rafts in VAMP3 KD cells is likely to be associated with an increase in FcϵRI phosphorylation. Even with the significant decrease in surface expression of FcϵRI in VAMP3 KD cells, the modified plasma membrane potential in relation to raft integrity is thought to affect FcϵRI signal transduction under Ag stimulation, resulting in enhanced distal gene transcription. Although further studies on the coalesced lipid raft domain and the efficacy of FcϵRI signal transduction are required, the enhanced FcϵRI signaling observed in VAMP3 KD cells could be attributed to the modulated plasma membrane caused by the absence of VAMP3.

MCs contain a variety of inflammatory mediators (e.g., histamine, cytokines, and chemokines) that can be released *via* exocytosis (e.g., degranulation) after activation. Inflammatory cytokines such as TNF-α and IL-6 are considered important contributors to MCs. Trafficking granules in MCs also intersect with endocytic routes to form specialized cytoplasmic granules called secretory lysosomes (46). Some mediators, such as histamine, reach granules *via* specific vesicular monoamine transporters directly from the cytoplasm [reviewed in (46)]. The comprehensive review summarizes the available data on granule biogenesis and signaling events that coordinate the complex steps leading to the release of inflammatory mediators from various vesicular carriers in MCs (46). The endocytic recycling under degranulation (i.e., β-hexosaminidase secretion) might be occurring in newly formed secretory granules within 180 min. For cytokine secretion, this has been generally observed not only in case of preformed mediator secretion (granule stored), but also in case of *de novo* synthesized mediators (47). In this study, detailed analysis of cytokine production and release up to 9 h following stimulation with Ag was performed. Results for the transcription and secretion levels of each cytokine for the selected differential time-courses indicated significantly slower transcriptional regulation of IL-6 compared to that of TNF-α (**Figure 5**). Furthermore, it has been suggested that inflammatory cytokines are differentially packaged in distinct secretory granules in macrophages (10). Manderson et al. showed that in macrophages, newly synthesized IL-6 accumulates in the Golgi complex and exits through the tubulovesicular carriers that deliver cargo proteins to the plasma membrane either as the sole labeled cargo or together with TNF-α, utilizing specific SNAREs to fuse with the recycling endosomes. Manderson et al. also demonstrated that there is compartmentalization of the cargo proteins within the recycling endosomes, wherein IL-6 is dynamically segregated from TNF-α and surface recycling transferrin (10). Thereafter, these cytokines are independently secreted, with TNF-α being delivered to phagocytic cups, but not IL-6. Therefore, they concluded that the recycling endosome plays a central role in orchestrating the differential secretion of cytokines during inflammation (10, 46). In MCs, however, precise mechanisms regarding the differential regulation of secretory responses have not been fully elucidated, especially in the context of SNARE involvement. A previous report on the function of VAMPs in MCs demonstrated that VAMP3 colocalizes with TNF-α (48). In our observations, microscopic images showed that IL-6 and TNF-α were distributed in distinct granules in MCs. Therefore, combined with the previous report and our findings, there are distinct granules packaging TNF-α and IL-6. VAMP3 mediated granule to granule fusion appears to be involved in Ag-induced secretion of TNF-α, but not IL-6, as shown in **Figure 5**. In addition, our RT-PCR analysis of several inflammatory cytokines also indicated differential regulation depending on VAMP3 expression; therefore, there might be a more complicated secretory regulation of cytokines in MCs in the context of SNARE distribution.

While this study indicated the multifunctional regulation of VAMP3 in MCs, there seem to be important limitations in the FcϵRI behavior (e.g., formation of FcϵRI clusters, interaction with lipid rafts) in Ag-stimulated MCs, which may explain the enhancement of FcϵRI signaling in VAMP3 KD cells. In addition, whether VAMP3 is involved in FcϵRI transport to the plasma membrane and the FcϵRI recycling pathway remains to be seen. Further investigation into this problem may facilitate a deeper understanding of the role of VAMP3 in allergic responses to MC. Additionally, compensation mechanisms involving SNAREs are also involved in SNARE-mediated secretion of inflammatory mediators in MCs, which might lead to complex secretory responses. This study also showed that the marked knockdown of VAMP3 did not result in the complete inhibition of β-hexosaminidase release (**Figure 1** **)**. A study using VAMP8-deficient mice indicated that VAMP3-containing SNARE complexes were increased in MCs from VAMP8-deficient cells. In VAMP8-deficient MCs, constitutive vesicular trafficking is perturbed by increased SNARE complex formation between VAMP3 and SNAP23 (49). Thus, the orchestrated mechanisms of SNAREs may be responsible for FcϵRI-mediated secretory responses in MCs.

In conclusion, our study demonstrates the multifaceted role of VAMP3, which appear to be involved in the heterogeneity of MC secretory responses. The results of this study may provide a foundation for identifying novel therapeutic targets in the treatment of allergic reactions to MCs.

## Data availability statement

The original contributions presented in the study are included in the article/**Supplementary Material**. Further inquiries can be directed to the corresponding author.

## Author contributions

SM, MS, HK, and YN contributed by conducting experiments, summarizing data, and/or generating the necessary reagents. YN and RS conceived of and directed the study. SM, MS, HK, YN, and RS participated in the writing of the manuscript. All authors contributed to the article and approved the submitted version.

## Funding

This research was funded by JSPS KAKENHI, grant numbers 20K15992 (to YN), 16H05082, and 19H03369 (to RS). This research was also funded by the Uehara Memorial Foundation, the Naito Foundation, and the Takeda Science Foundation (to RS).

## Conflict of interest

The authors declare that the research was conducted in the absence of any commercial or financial relationships that could be construed as a potential conflict of interest.

## Publisher’s note

All claims expressed in this article are solely those of the authors and do not necessarily represent those of their affiliated organizations, or those of the publisher, the editors and the reviewers. Any product that may be evaluated in this article, or claim that may be made by its manufacturer, is not guaranteed or endorsed by the publisher.

## Abbreviations

MC, Mast cell; Ag, antigen; Ig, immunoglobulin; FcϵRI, high affinity receptor for IgE; DNP, 2,4-dinitrophenyl; HSA, human serum albumin; SNARE, soluble N-ethyl-maleimide-sensitive factor attachment protein receptor; VAMP, vesicle-associated membrane protein; SNAP, soluble N-ethylmaleimide-sensitive fusion protein; KD, knockdown; TNF-α, tumor necrosis factor alpha; IL, interleukin; shRNA, short hairpin RNA; NT, non-target.
