# Supplementary material for: Multifunctional regulation of VAMP3 in exocytic and endocytic pathways of RBL-2H3 cells
Source: Front Immunol. 2022 Aug 5;13:885868. doi: 10.3389/fimmu.2022.885868 (PMC9388853; doi:10.3389/fimmu.2022.885868)
Supplement: Supplementary file 1 [file DataSheet_1.docx]

Supplementary Material

**Supplementary tables**

| Primer | Forward (5′➝3′) | Reverse (5′➝3′) | RE _(For)_ | RE _(Rev)_ |
| --- | --- | --- | --- | --- |
| CD63 | CCGCTCGAGATGGCGGTGGAAGGAGGAA | CCCAAGCTTCATTACTTCGTAGCCACTCC | XhoⅠ | HindⅢ |
| VAMP3 | ACGCGTCGACATGTCTACAGGGGTGCCTTC | CGCGGATCCTTAAGAGACACACCACAC | SalⅠ | BamHⅠ |

**Supplementary Table 1.** Sequences of primers used for plasmid construction

| Primer | Forward (5'→3') | Reverse (5'→3') |
| --- | --- | --- |
| VAMP3 | CGCCGCCAAAATGTCTAC | TGTCCACCTCATCTACTTG |
| TNF-α | CAGATGGGCTGTACCTTATC | GGTATGAAATGGCAAATCGG |
| CCL2 | CTACTCATTCACTGGCAAGA | TCTTGAGCTTGGTGACAAAT |
| IL-2 | GCAGGCCACAGAATTGAAAC | CCAGCGTCTTCCAAGTGAA |
| IL-4 | CAGGGTGCTTCGCAAATTTTAC | ACCGAGAACCCCAGACTTGTT |
| IL-6 | TCCTACCCCAACTTCCAATGCTC | GGTCCTTAGCCACTCCTTCTGT |
| IL-13 | ATCACACAAGACCAGAAGACTTC | AACTGGGCTACTTCGATTTTGG |
| FcεRIα | ACCGTGGATTAGAATACTTACAGGAG | CGTCAGCAGAAGATTGGAGCAG |
| FcεRIβ | GCAAAAGCTCTACCAGAGAA | CTACGCTCAAATTCTTGTCC |
| FcεRIγ | CCCTGCTCTACTGTCGACTCA | TCTCACGGCTGGCTATGTCT |
| GAPDH | GACAACTTTGGCATCGTGGA | ATGCAGGGATGATGTTCTGG |

**Supplementary Table 2.** Sequences of primers used for gene expression analysis by qPCR.

**Supplementary Figures**

**
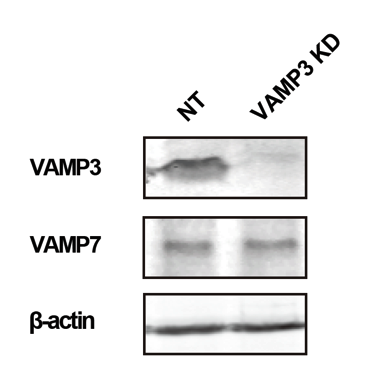
**

**Supplementary Figure 1.** The expression levels of VAMP7 were not altered in VAMP3 KD cells.

Cell lysates were subjected to western blot analysis using anti-VAMP3 antibody and anti-VAMP7 antibody followed by fluorescent conjugated secondary antibodies; images were obtained using Odyssey scanner. NT: control knockdown with non-targeting shRNA plasmid.

**
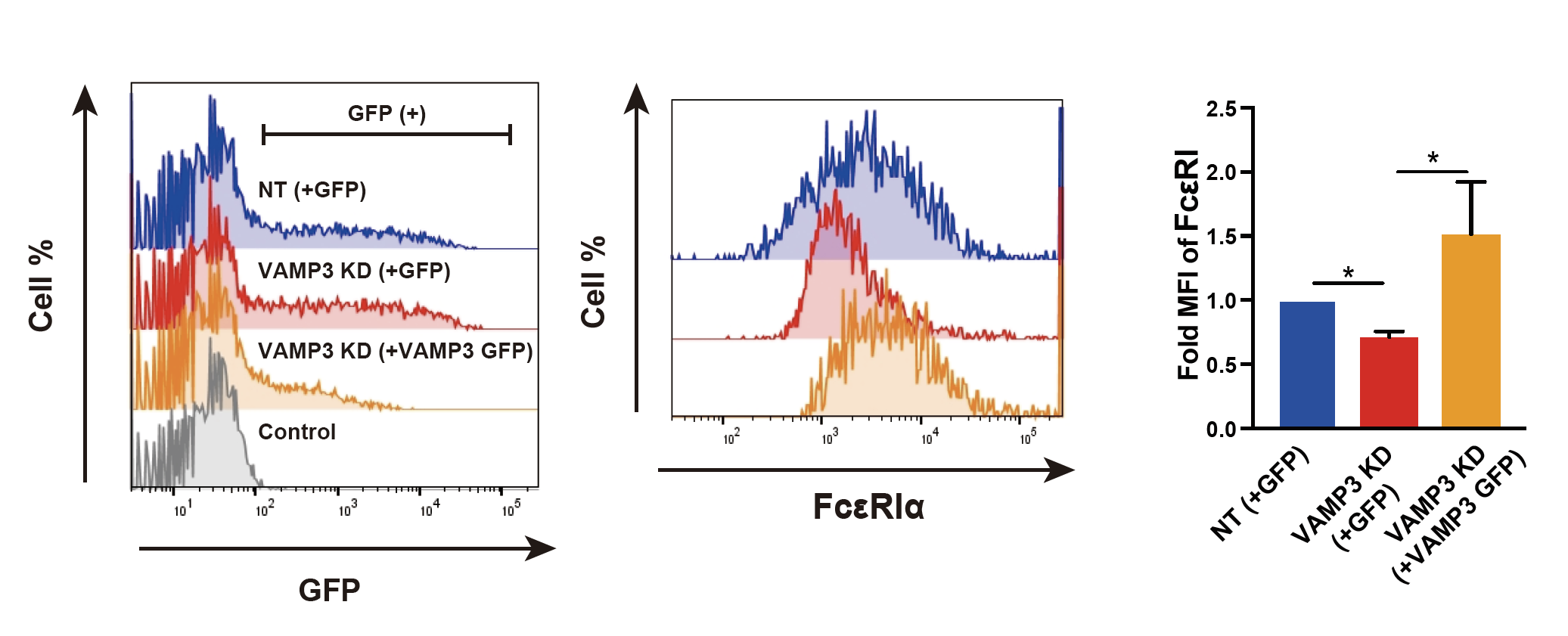
**

**Supplementary Figure 2.** Flow cytometric analysis of VAMP3 rescued cells.

Cells were fixed and treated with Alexa Fluor 647-conjugated anti FcεRIα antibody 24 h after GFP-tagged VAMP3 transfection and GFP-positive cells (left histogram) were analyzed to quantify FcεRI expression (right histogram). The transfection and FACS analysis were conducted as described in the main text. Bar graph shows fold change and mean fluorescence intensity (MFI) of FcεRIα. n = 4 independent experiments and representative histograms are shown. NT: control knockdown with non-targeting shRNA plasmid. *p<0.05


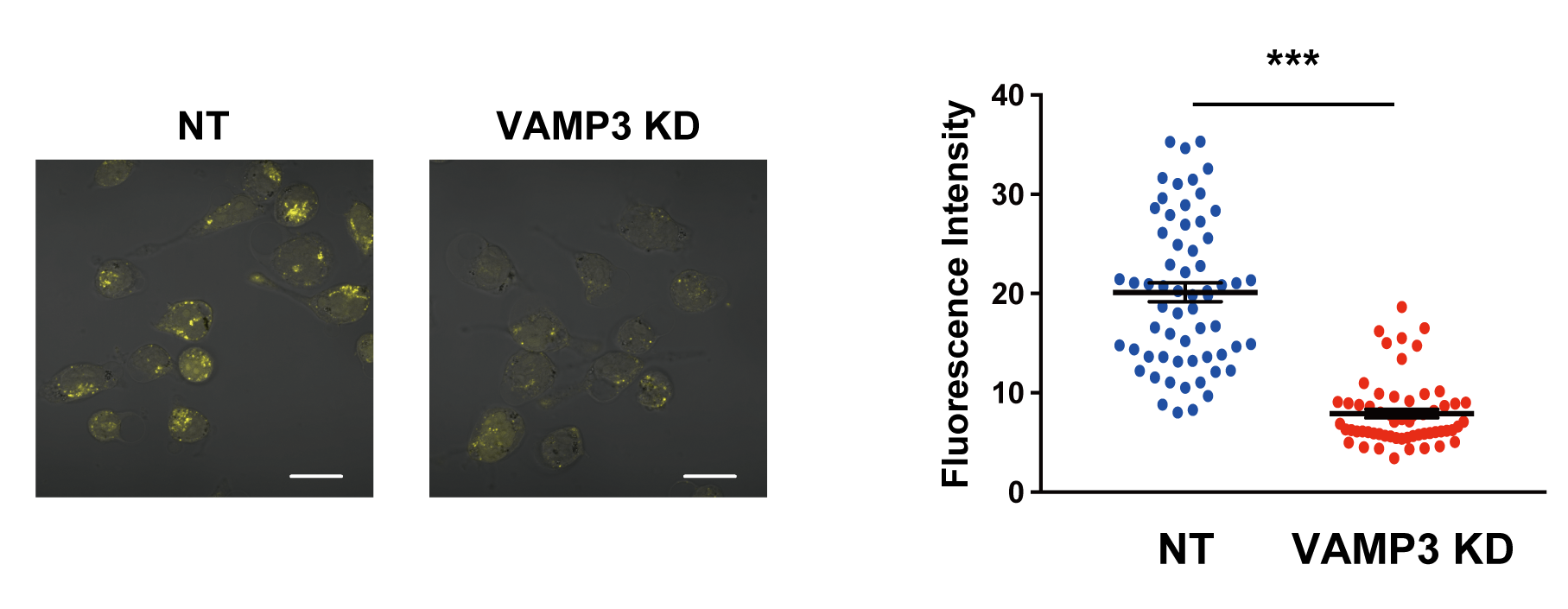


**Supplementary Figure 3.** Endocytosis measured using Lucifer Yellow uptake.

IgE-loaded cells were stimulated with Ag (50 ng/ml) in Lucifer Yellow dye containing Tyrode’s buffer for 3 h (magnification ×40, scale bar = 20 μm). The graph on the right shows the fluorescence intensity of Lucifer Yellow per cell. Data were obtained from 59 (NT) and 62 (VAMP3 KD) cells and are presented as the mean ± SE. NT: control knockdown with a nontargeting shRNA plasmid. ***p<0.001


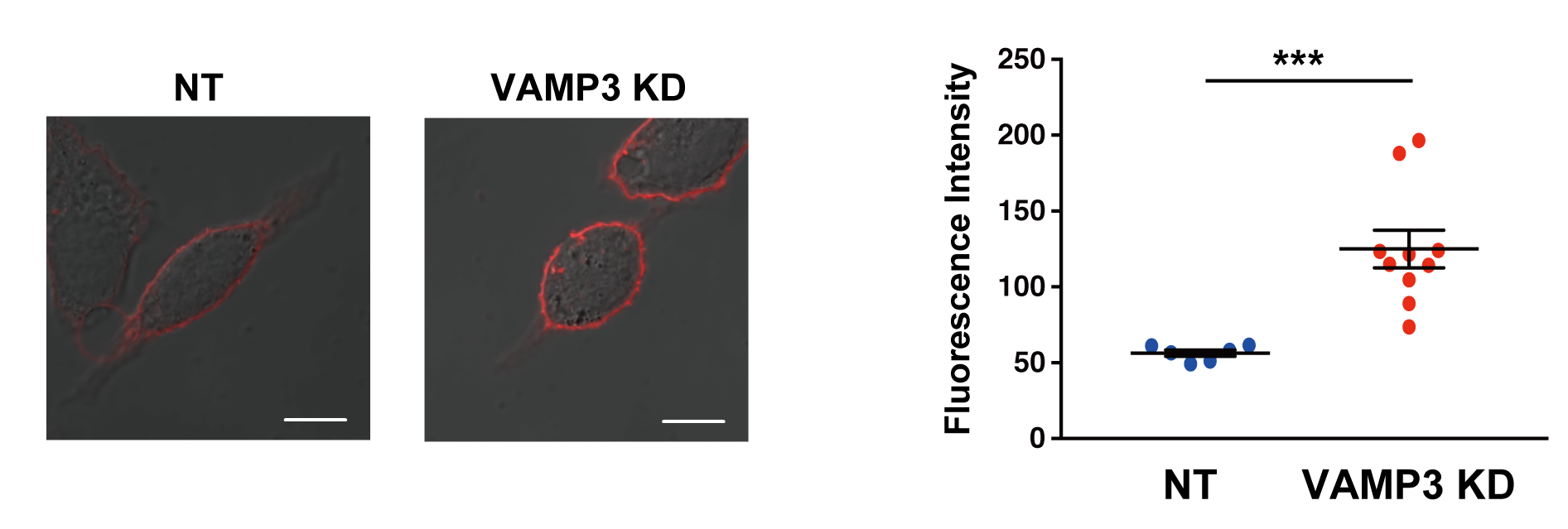


**Supplementary Figure 4.** Lipid raft content in resting cells was measured using CTxB.

Cells were labeled with CTxB-Alexa Fluor 647 to illustrate cell surface expression of GM1, a marker of lipid rafts, and monitored by confocal microscopy (magnification ×63, scale bar = 10 μm). The graph on the right shows fluorescence intensity of the membrane per cell. Data were obtained from 6 (NT) to 10 (VAMP3 KD) cells and are presented as the mean ± SE. NT: control knockdown with a nontargeting shRNA plasmid. ***p<0.001; ns, not significant


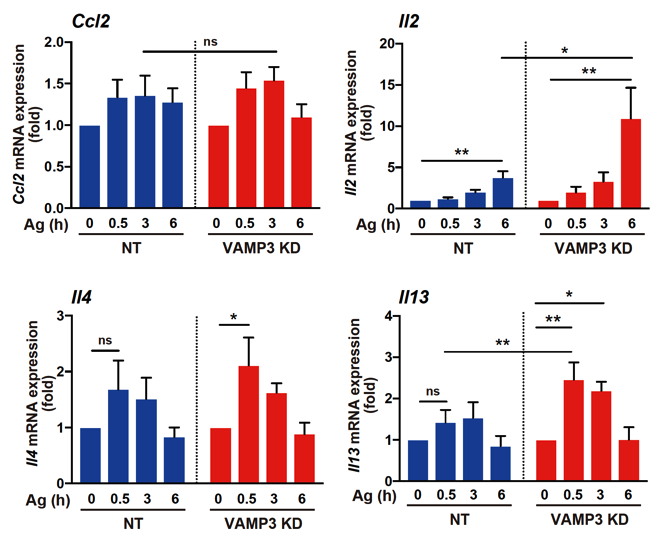


**Supplementary Figure 5.** Cytokine gene expression was measured using RT-PCR.

Time-dependent mRNA expression of CCL2, IL-2, IL-4, and IL-13 following Ag stimulation (50 ng/ml) were compared to NT cells and VAMP3 KD cells. The RT-PCR and statistical analysis were conducted as described in the main text. n = 5–7 independent experiments. *p<0.05, **p<0.01; ns, not significant

**Supplementary Experimental Procedures**

*Lucifer Yellow dye assay*

Anti-DNP IgE 1 µg/ml loaded cells were stimulated with 50 ng/mL DNP-HSA and 1 mg/mL Lucifer Yellow (Invitrogen, Carlsbad, CA) in Tyrode’s buffer for 3 h. The stimulated cells were fixed with 4% paraformaldehyde and subjected to confocal laser scanning microscopy. The average fluorescence intensity was measured using MIPAV (NIH, Bethesda, MD, USA).

*Lipid raft staining*

Resting cells were fixed with 4% paraformaldehyde, incubated with cholera toxin subunit-Alexa Fluor 647 (Invitrogen, Carlsbad, CA, USA) for 15 min, and subjected to confocal laser scanning microscopy. Average fluorescence intensity was measured using MIPAV (NIH, Bethesda, MD, USA).

*Plasmid design*

Amplification of the respective genes (CD63 and VAMP3) for plasmid construction was conducted using PCR with the corresponding primer sets (Supplemental Table 2). The amplified DNA fragments were inserted into the respective expression vectors using the restriction enzymes XhoI and HindIII (for pTagGFP2-N, NEB, Ipswich, MA, USA) and SalI and BamHI (for pTagRFP-C, NEB) using a DNA ligation kit (TaKaRa, Shiga, Japan) according to the manufacturer's instructions.
